# *Caenorhabditis elegans*: A Useful Model for Studying Metabolic Disorders in Which Oxidative Stress Is a Contributing Factor

**DOI:** 10.1155/2014/705253

**Published:** 2014-05-18

**Authors:** Elizabeth Moreno-Arriola, Noemí Cárdenas-Rodríguez, Elvia Coballase-Urrutia, José Pedraza-Chaverri, Liliana Carmona-Aparicio, Daniel Ortega-Cuellar

**Affiliations:** ^1^Laboratory of Experimental Nutrition, National Institute of Pediatrics, 04530 Mexico City, DF, Mexico; ^2^Laboratory of Neurochemistry, National Institute of Pediatrics, 04530 Mexico City, DF, Mexico; ^3^Department of Biology, Faculty of Chemistry, University City, UNAM, 04150 Mexico City, DF, Mexico

## Abstract

*Caenorhabditis elegans* is a powerful model organism that is invaluable for experimental research because it can be used to recapitulate most human diseases at either the metabolic or genomic level *in vivo*. This organism contains many key components related to metabolic and oxidative stress networks that could conceivably allow us to increase and integrate information to understand the causes and mechanisms of complex diseases. Oxidative stress is an etiological factor that influences numerous human diseases, including diabetes. *C. elegans* displays remarkably similar molecular bases and cellular pathways to those of mammals. Defects in the insulin/insulin-like growth factor-1 signaling pathway or increased ROS levels induce the conserved phase II detoxification response via the SKN-1 pathway to fight against oxidative stress. However, it is noteworthy that, aside from the detrimental effects of ROS, they have been proposed as second messengers that trigger the mitohormetic response to attenuate the adverse effects of oxidative stress. Herein, we briefly describe the importance of *C. elegans* as an experimental model system for studying metabolic disorders related to oxidative stress and the molecular mechanisms that underlie their pathophysiology.

## 1. Introduction

Diabetes mellitus is a metabolic disorder that affects millions of people worldwide and contributes considerably to global mortality [1]. Diabetes is characterized by poor control of glucose homeostasis. Although many advances have been made in understanding the pathophysiology of diabetes mellitus, its prevalence continues to increase, in part because of lifestyle changes and increased overall life expectancy. Diverse studies have demonstrated that oxidative stress participates in the progression of diabetes complications [2–4]. This progression occurs in part because high glucose concentrations in diabetes lead to glucose oxidation via the tricarboxylic acid cycle (TCA), which in turn generates electron donors (NADH, FADH_2_) for the respiratory chain (RC) that consequently induce the overproduction of reactive oxygen species (ROS). This ROS overproduction triggers an adverse response by modulating several metabolic and signaling pathways, exacerbating diabetic complications [5]. Therefore, there is an urgent need for new approaches for the prevention and treatment of this disease. Most studies have been performed using rodent models of type 1 or type 2 diabetes in which hyperglycemia is induced via genetic, pharmacological, or dietary manipulation. However, there is a huge knowledge gap between the pathogenic mechanisms that cause diabetic complications and the treatments, which prevents the development of appropriate therapeutic interventions. Improving the understanding of the mechanisms that modulate the numerous metabolic pathways in humans requires studies using model organisms that recapitulate most aspects of human disease at either the phenotypic or genomic level* in vivo*. The worm* C. elegans* represents a relevant model for elucidating the metabolic regulation mechanisms at the molecular level, matching or even improving upon the available mammalian model. Throughout this review, we will provide evidence for similarities between* C. elegans* and mammals that could contribute to the elucidation of the molecular pathways that are implicated in human diseases.

## 2. Oxidative Protection System in* C. elegans*

### 2.1. Overview of* C. elegans*

The number of studies that use* C. elegans* as a model system has grown significantly in recent decades. Interest in this nematode has suddenly soared for many reasons. First,* C. elegans* was the first animal for which the genome was completely sequenced [6]. Second, it is estimated that more than half of* C. elegans* genes are homologous to genes implicated in human diseases [7, 8]. Third, this model organism is maintained under simple experimental conditions in the laboratory and has an optically transparent body that is amenable to the use of fluorescent probes to visualize oxidative stress within the nematodes* in vivo* [9, 10].* C. elegans* contains many cell types that represent the major tissues of complex metazoans, such as muscle, intestinal, nervous, and epithelial tissue [11]. However, caution is warranted because this organism exhibits important differences from mammals; for example, it does not possess a circulatory system, which could limit its utility as a model for some diseases. Despite this difference,* C. elegans* shares many similarities with mammals, including signaling pathways, such as the insulin/insulin-like growth factor-1 signaling (IIS) pathway [12]. In summary,* C. elegans* is a very useful system for studying the organismal integration of the oxidative stress response [13].

### 2.2. Aspects of the Mitochondrial System Shared between Mammals and* C. elegans*

In mammals, the principal metabolic machinery that produces energy from nutrients is the mitochondrion, which drives ATP formation throughout the entire body via oxidative phosphorylation (OXPHOS), a system that comprises several redox reactions in the RC that are coupled to ATP synthesis [14]. Typically, the mitochondrial RC, which is the final pathway of OXPHOS, consists of five macromolecular enzymatic complexes (I–V) that catalyze the transfer of electrons from reducing equivalents (NADH or succinate) from the Krebs cycle through the chain [15]. These proteins have been highly conserved throughout evolution [16] and include NADH-coenzyme Q (CoQ; also referred to as ubiquinone) reductase (complex I); succinate-CoQ reductase (complex II); CoQ-cytochrome c reductase (complex III); cytochrome c oxidase (complex IV); and ATP synthase (complex V). Ubiquinone and cytochrome c are two freely diffusible molecules that mediate the transfer of electrons between the complexes [17].

In lower eukaryotes, such as* C. elegans*, the function of the electron transport system, its size, and its genetic contents are similar to those of mammals [18, 19]. Recently, Li et al. described the use of a shotgun proteomic approach to identify mitochondrial proteins in* C. elegans*, finding that 405* C. elegans* mitochondrial proteins possess human homologs, indicating a relatively high conservation of mitochondrial proteins between eukaryotic organisms [20]. Interestingly, mutations in several mitochondrial components (collectively referred to as Mit mutants) can disrupt the mitochondrial electron transport chain and exert various effects on the life expectancy of* C. elegans*. A mutation of* nuo-1*, which encodes for mitochondrial complex I, exhibits hallmark characteristics of complex I dysfunction in the mammalian system. These properties include lactic acidosis, decreased mitochondrial respiration, hypersensitivity to exogenous oxidative stressors (hyperoxia or paraquat), and decreased lifespan, conceivably due to elevated oxidative stress [21]. Conversely, mutations of the* isp-1* and* nuo-6* genes that encode subunits of complexes I and III, respectively, increase the lifespan without affecting the ATP levels [22]. In general, the information available to date indicates that the RC metabolism and bioenergetics of the nematode are very similar to those of mammals, and several pathways of intermediary energy metabolism are also conserved in* C. elegans* [18, 20, 23–25].

## 3. Machinery against Oxidative Stress in* C. elegans*

**Figure 1:**
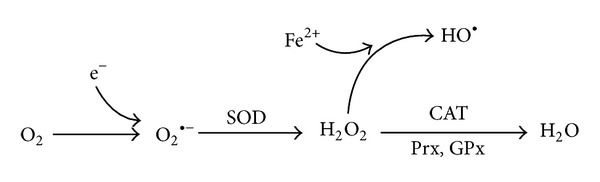
Machinery that protects against oxidative stress and intracellular ROS overproduction. The principal ROS include the superoxide anion (O_2_^•−^), hydrogen peroxide (H_2_O_2_), and the hydroxyl radical (HO^•^). Cellular redox homeostasis is maintained by a set of antioxidant enzymes, such as superoxide dismutase (SOD), catalase (CAT), glutathione peroxidase (GPx), and peroxiredoxin (Prx).

Aside from its bioenergetic activity, the OXPHOS system is understood to be the major endogenous source of cellular ROS [26, 27], thus contributing to mitochondrial damage and potentially triggering diverse pathologies related to redox signaling [28]. Therefore, a basic conception of how mitochondria produce ROS and how these molecules function is vital for understanding a range of currently important pathologies. The principal ROS include the superoxide anion (O_2_^•−^), hydrogen peroxide (H_2_O_2_), and the hydroxyl radical (HO^•^). O_2_^•−^ is typically the primary ROS species and is generated via the interaction between an oxygen molecule and NADPH oxidase or other components (flavines, quinones, and thioles) and contributes to cell damage via oxidative stress [29]. Cellular redox homeostasis is maintained by a set of delicate balances between ROS production and the antioxidant system. Numerous antioxidant enzymes, such as superoxide dismutase (SOD), catalase (CAT), glutathione peroxidase (GPx), and peroxiredoxins (Prxs), have been identified to defend against ROS overproduction [30–32] (Figure 1).

### 3.1. SOD

In mammals, SOD family enzymes represent the first line of antioxidant defense against ROS. SOD converts O_2_^•−^ to H_2_O_2_, which can subsequently be converted to water by CAT, GPx, or Prxs [33]. SOD is the only enzyme that can detoxify superoxide [34] and is found in various cellular compartments. SOD1 (Cu/ZnSOD) is the predominant superoxide scavenger and is localized in the cytoplasm, the mitochondrial intermembrane space, the nucleus, and lysosomes. SOD2 (MnSOD) and SOD3 are localized in the mitochondria and the extracellular matrix, respectively [29].

Similar to mammals,* C. elegans* possesses six SOD isoforms; two are mitochondrial (known as MnSODs) and are encoded by the* sod-2 *and* sod-3* genes; two are cytosolic (Cu/ZnSODs) and are encoded by* sod-1* and* sod-5*; and two are predicted to be extracellular Cu/ZnSOD isoforms, both of which are encoded by* sod-4* (Table 1).* sod-2* and* sod-1* are highly expressed during normal development.* sod-3* and* sod-5* are minor isoforms whose expression levels are increased during the dauer stage [35, 36]. Several research groups have eliminated the expression of individual* sod* genes and have found that deletion of each gene displays little or no detrimental effect on the* C. elegans* lifespan [37–40]. More recently, Van Raamsdonk and Hekimi demonstrated that* C. elegans* containing quintuple mutations of genes* sod-1, sod-2, sod-3, sod-4*, and* sod-5* (*sod-12345*) exhibited a normal lifespan, but SOD activity was also required to survive acute stressors. Additionally, their results indicate that superoxide not only is a toxic byproduct of metabolism but also is involved in a ROS-mediated signaling mechanism that can result in increased longevity. The same study also questions the notion that oxidative stress is the primary cause of aging [41].

### 3.2. CAT

CAT is a H_2_O_2_ oxidoreductase heme-containing enzyme that removes H_2_O_2_ to generate oxygen and water during oxidative stress. Increased CAT activity helps to overcome the damage to tissue metabolism by reducing the toxic levels of H_2_O_2_ [53].

The* C. elegans* genome possesses three catalase genes, encoding* ctl-1, ctl-2*, and* ctl-3* [54]*. ctl-1* is cytosolic, whereas* ctl-2* and* ctl-3* are peroxisomal (Table 1).* ctl-1* and* ctl-2* play roles in the organismal lifespan [44, 45], whereas* ctl-3* remains uncharacterized. Similar to SOD, the functions of catalases in the lifespan are ambiguous because the loss of* ctl-2* shortens the lifespan [44]; however, other findings indicate that oxidative stress induced by dietary restriction increases catalase activity [55].

### 3.3. GPx

GPx is the general term for a family of multiple isozymes (GPx1–8) that catalyze the reduction of H_2_O_2_ or oxidized lipids to water using glutathione (GSH) as an electron donor [49, 56].

As in humans,* C. elegans* contains several genes corresponding to GPx (*gpx-1* to* gpx-8*), but limited data are available regarding these proteins.* gpx-1* (F26E4.12) encodes a phospholipid hydroperoxide GPx that is a homolog of human GPx4. The* gpx-1* enzyme is predicted to catalyze the reduction of phospholipid hydroperoxides using glutathione because loss of* gpx-1* activity via RNAi results in increased cellular levels of the unsaturated aldehyde 4-hydroxynonenal (4-HNE), a lipid peroxidation product [50].

### 3.4. GST

GSTs are another set of cellular detoxification enzymes that catalyze the conjugation of exogenous and endogenous compounds to GSH to prevent oxidative stress-induced injury [57].

The* C. elegans* genome contains over 50 putative GSTs [52]. Of these, three GSTs, referred to as* Ce-*GST-p24* (K08F4.7), CeGSTP2-2 (gst-10)*, and* GSTO-1 (C29E4.7)*, modulate the oxidative stress response (Table 1) [52]. In fact, the separate overexpression of each of these three genes led to an increased resistance to some stress inducers, such as juglone, paraquat, and cumene hydroperoxide, and silencing of these GSTs via RNAi resulted in increased sensitivity to the aforementioned prooxidant compounds [58–60].

### 3.5. Prxs

Prxs are a large ubiquitous family of proteins that possess cysteine-containing redox active centers [61, 62] that use peroxidatic cysteine to reduce hydroperoxides and release water. These enzymes are classified according to their cysteine contents: there are one-cysteine (Prx VI) and two-cysteine peroxiredoxins (I–IV, V). Prxs function as a signal regulator at specific locations by modulating the local ROS levels in a redox-dependent manner [29].

The* C. elegans* genome includes three Prx genes: prdx-2, prdx-3, and prdx-6 (which should not be confused with the prx, or peroxisomal membrane protein, genes); however, only two of these genes have been studied. PRDX-2 is an enzyme that protects against the toxic effects of H_2_O_2_; however, it is also noteworthy that the loss of this protein triggers increased resistance to oxidative stress apparently via a signaling mechanism that increases the levels of other antioxidants and phase II detoxification enzymes [48] in a manner that is independent of the SKN-1 pathway, mentioned later. Nonetheless, despite their increased resistance to some forms of oxidative stress,* prdx-2* mutant animals are short-lived, suggesting that* prdx-2* may promote longevity and protect against environmental stress via distinct mechanisms [48]. However, no effect on oxidative stress has been detected for* prdx-3*, but it does display mitochondrial uncoupling activity, suggesting its importance in energy metabolism [47].

## 4. Oxidative Stress and Disease

Given the wide spectrum of oxidative species that are generated in the cell, it is clear that many biomolecules (DNA, proteins, lipids, and others) are vulnerable to ROS attack. This damage may lead to or exacerbate several metabolic diseases. Despite its evolutionary distance from mammals,* C. elegans* represents an adequate model to complement both* in vitro* and* in vivo* vertebrate models of oxidative stress. This model system has provided insight into the molecular mechanisms of signal transduction pathways, such as the oxidative stress and IIS pathways, that influence numerous human diseases, including diabetes [63, 64]. In diabetic patients, some metabolic abnormalities include mitochondrial O_2_^•−^ overproduction and increased formation of advanced glycation end products (AGEs) and lipid peroxidation of low-density lipoprotein (LDL) via a superoxide-dependent pathway, resulting in several effects that are toxic to organisms [65, 66]. Similar to mammals, Schlotterer et al. recently proposed that* C. elegans* represents a suitable organism to study glucose toxicity because exposing nematodes to high glucose concentrations exerts detrimental effects on longevity, increasing ROS production and AGE modification of mitochondrial proteins in an insulin pathway-independent manner [67].

### 4.1. Nuclear Factor E2-Related Factor (NRF-2) Signaling Pathways Defend against Oxidative Stress and Metabolic Diseases

Eukaryotic cells possess Nrf-2 signaling pathways that defend against oxidative stress by inducing the expression of phase II detoxification genes [68, 69]. Nrf-2 is a basic leucine zipper-containing transcription factor that binds to antioxidant response element (ARE) sequences in the promoter regions of specific genes to modulate the antioxidant response system [70]. Under normal physiological conditions, Nrf-2 is inactivated by binding to Kelch-like ECH-associating protein 1 (Keap1) in the cytoplasm. However, under oxidative stress, Nrf-2 is released from Keap1 and is translocated to the nucleus, where it binds to ARE and transactivates genes corresponding to detoxifying and antioxidant enzymes, such as *γ*-glutamyl cysteine ligase (*γ*-GCL), the cystine/glutamate antiporter (xCT), *μ*-GST, heme oxygenase-1 (HO-1), and others [71–73]. Additionally, Nrf-2 has been suggested to be involved in energy-related pathologies, such as diabetes and obesity [74]. For instance, streptozotocin-induced diabetic mice lacking the Nrf-2 gene exhibit increases in both oxidative stress and blood glucose levels, most likely via the enhanced mRNA expression of gluconeogenic genes (glucose-6-phosphatase and phosphoenolpyruvate carboxykinase) and the diminished expression of glycolytic genes (pyruvate kinase) [74]. Similar to diet-induced obese mice, the activation of Nrf-2 using oltipraz, a synthetic dithiolethione, triggers the regulation of detoxifying enzymes via Nrf-2 [75], improving insulin resistance and obesity and reducing oxidative stress [76]. In addition, constitutive Nrf-2 activation inhibited lipid accumulation in white adipose tissue, suppressed adipogenesis, induced insulin resistance and glucose intolerance, and increased hepatic steatosis in Lep (ob/ob) mice [77]. Thus, it is hypothesized that the transcription factor Nrf2, in addition to its role in protecting organisms against oxidative stress, may be a critical target for preventing diabetes mellitus.

### 4.2. Oxidative Stress Resistance via the SKN-1 and IIS Pathways in* C. elegans*

**Figure 2:**
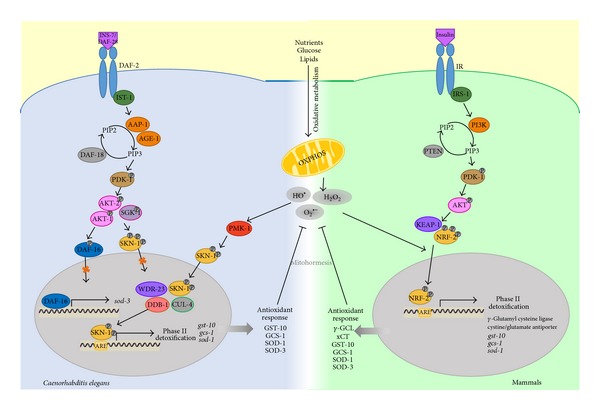
Cross-talk between mitochondrial metabolism and the IIS pathway is required to trigger the mitohormetic response in* C. elegans*. The* C. elegans* IIS pathway contains components that are nearly identical to those of mammals [87]; under conditions of nutrient supply, the IIS pathway is initiated by the binding of DAF-28 or INS-7 [88, 89] to DAF-2 [12, 90], subsequently triggering a cascade of phosphorylation events to activate specific kinases that inactivate the transcriptional factor DAF-16 and its target genes (e.g.,* sod-3*) [86, 91]. A similar mechanism occurs for the transcriptional factor SKN-1 via the kinases AKT-1/2 and SGK-1. Conversely, the transcriptional activity of SKN-1 is augmented by some stressors, such as oxidative stress, as a consequence of OXPHOS activity via PMK-1 kinase, culminating in the nuclear translocation of SKN-1 and its interaction with the DNA-binding sites (AREs) of its target genes (*gst-10*,  * gcs-1*, and* sod-1*). Finally, an antioxidant response is activated to prevent ROS-mediated cellular damage, which may support the mitohormetic theory. DAF-28 and INS-7, insulin-like peptides; DAF-2, insulin/IGF-1 receptor; IST-1, insulin receptor substrate 1 ortholog; AGE-1 and AAP-1, phosphatidylinositol 3-kinases; PIP2, phosphatidylinositol (4,5)-bisphosphate; PIP3, phosphatidylinositol-3,4,5-trisphosphate (PtdIns(3,4,5)P3); PDK-1, 3-phosphoinositide-dependent kinase 1; DAF-18, homologous to human PTEN; AKT1/2 and SGK-1, orthologs of the serine/threonine kinase Akt/PKB; DAF-16, FOXO transcription factor; SKN-1, skinhead family member 1, the ortholog of mammalian Nrf-2; PMK-1, the p38 MAPK ortholog; WDR-23, possible functional homolog of Keap1; DDB-1/CUL-4, ubiquitin ligase complex; ARE, antioxidant response element; OXPHOS, oxidative phosphorylation; GST-10/gst-10, glutathione S-transferase-10; GCS-1/*gcs*-1, *γ*-glutamyl cysteine synthetase-1; and SOD-1/sod-1 and SOD-3/*sod-3*, superoxide dismutase-1 and -3, respectively.

In* C. elegans*, SKN-1 is the ortholog of mammalian Nrf-2 and is also activated in response to elevated levels or oxidative stress or to compounds such as H_2_O_2_, paraquat, and juglone via the induction of phase II detoxification gene transcription [78, 79]. In fact, it is suggested that the increased lifespan of* C. elegans* under caloric restriction occurs due to SKN-1 activation via increased stress tolerance resulting from reduced IIS pathway activity [80, 81]. The regulation mechanisms of SKN-1 are divergent from those of mammals because* C. elegans* does not possess a recognizable Keap1 protein. Alternatively,* C. elegans* expresses WDR-23, a protein that acts as a homolog of Keap1, by promoting the nuclear translocation [82] and binding of SKN-1 to ARE sites [83]. SKN-1 activity is regulated by phosphorylation. Thus, under basal conditions inhibitory phosphorylation by AKT-1/2 and SGK-1 (key components of the IIS pathway) inactivates SKN-1 by maintaining it in the cytosol [79]. Conversely, under conditions of oxidative stress or reduced IIS pathway activity (induced by caloric restriction), the PMK-1 protein (ortholog of the mammalian p38 MAPK) phosphorylates SKN-1 to promote its nuclear translocation and subsequently induce the transcription of phase II detoxification genes, such as* gcs-1* (encoding *γ*-glutamyl cysteine synthetase),* gst-10* (encoding isoforms of glutathione S-transferase), and* sod-1* (encoding SOD) [79, 84, 85] to generate the ROS response (Figure 2). Another ROS protection mechanism is conferred by DAF-16 via the IIS pathway, which regulates MnSOD gene expression [86]. Evidence has demonstrated that defects in the IIS pathway change the level of cellular energy metabolism (e.g., glucose uptake) and activate DAF-16, increasing the gene expression of* sod-3*, which in turn triggers the activity of this mitochondrial antioxidant system [81] (Figure 2). Therefore, based on the above findings,* C. elegans* represents a useful model organism to study the roles of the SKN-1 and IIS pathways as master regulators of the cellular defense system against oxidative stress.

## 5. The Roles of ROS as Second Messengers (Mitohormesis) and in Disease

As mentioned above, ROS have been associated with cellular damage. However, diverse studies have challenged the concept of ROS as simply detrimental; instead, they have been proposed as second messengers that trigger a program of transcriptional and metabolic shifts that initiate an adaptive ROS signaling response to attenuate the adverse effects of oxidative stress [92, 93]. Positive effects of ROS have been detected in both humans and* C. elegans*. Healthy young men undergoing physical exercise efficiently increased ROS production and thus counteracted insulin resistance [94]. These findings are consistent with the concept of mitohormesis, in which exercise-induced oxidative stress causes an adaptive response to promote the endogenous antioxidant defense [94]. This adaptive response to ROS, often referred to as mitochondrial hormesis, the hormetic response, or mitohormesis [95], results in compensatory biological processes following an initial disruption of homeostasis [96]. Similar effects have been demonstrated in* C. elegans* (Figure 2); when this nematode is in an active growth and nutrient consumption state, elevated ROS levels activate its stress response and delay aging [97].

ROS have been suggested as second messengers under mild stress conditions, which in turn enhance vitality, in part, because mitochondrial ROS production alters various signaling pathways, functioning as an alarm system that alerts cells to some stressors and responds in a manner that corresponds to the intensity of the detected damage. In fact, ROS appear to function as a signaling intermediate to facilitate cellular adaptation to some types of stress, although it remains to be clarified whether ROS are important for maintaining homeostasis in the absence of oxidative stress [49]. Accordingly, it is plausible that, in circumstances in which both mitochondrial activity and, hence, ROS production are augmented (e.g., diabetes or exercise), the hormetic response may be activated to increase ROS production, upregulating the expression of ROS-neutralizing enzymes, such as SOD2 and GPx. This response involves, in part, the transcription factor SNK-1/Nrf-2, which promotes the transcription of the aforementioned enzymes (Figure 2) [98]. It is noteworthy that the hormetic response may explain why, because of environmental risk factors or lifestyle, not all individuals predisposed to develop diabetes ultimately progress to overt disease. In summary, transiently increased levels of oxidative stress may improve rather than worsen the stress response, reflecting a potentially health-promoting process to prevent metabolic diseases, such as insulin resistance and diabetes, which is consistent with the concept of mitohormesis.

Historically, the principal theory regarding oxidative stress is that the ROS-induced accumulation of molecular damage significantly contributes to numerous human diseases, including diabetes, cardiovascular diseases, atherosclerosis, cancer, and aging [99]. The aforementioned characteristics of* C. elegans* render it a robust model system to investigate oxidative stress, and this model might contribute greatly to our understanding of the role of mitochondria and their integration into the oxidative stress network to regulate the health of the cell and organism.

## 6. Concluding Remarks

Despite extensive research using various model organisms, there are many unanswered questions concerning the connection between oxidative stress and the pathogenesis of metabolic diseases. Therefore, the development of animal models that serve as models of human diseases has ushered in the study of metabolic diseases. The* C. elegans* model system offers several distinct advantages, including its easy manipulation and maintenance in the laboratory and its high genetic homology to humans, that facilitate multiple studies at both the metabolic and molecular levels. Despite the important differences between nematodes and humans that must be considered, studies using* C. elegans* could significantly contribute to the knowledge gained from classical model organisms by facilitating the generation of results that cannot be produced using whole organisms* in vivo* and the identification of novel and rational treatments. Essentially, the findings from* C. elegans* described in this review indicate similar strategies to fight against oxidative stress, and* C. elegans* can conceivably be used as a powerful model system to delineate the genetic and molecular mechanisms that could be involved in human metabolic diseases, such as diabetes.

## Figures and Tables

**Table 1:** A comparison of evolutionarily conserved antioxidant enzymes expressed in mammals and *C. elegans*.

Mammals			*C. elegans *		
Enzyme	Cellular localization	Ref.	Enzyme	Cellular localization	Ref.
SOD1 (Cu/ZnSOD)	Cytosol Mitochondria Nucleus Lysosomes	[29]	Cu/ZnSODs (*sod-1* and *sod-5*)	Cytosol	[35–37, 42]
SOD2 (MnSOD)	Mitochondria	[29]	MnSODs (*sod-2 *and *sod-3*)	Mitochondria	
SOD3	Extracellular matrix	[29]	Predicted Cu/ZnSOD (*sod-4*)	Extracellular matrix	

Catalase	Cytosol Peroxisomes	[43]	*ctl-1 *	Cytosol	[44, 45]
			*ctl-2*, *ctl-3 *	Peroxisomes	

Peroxiredoxins (PrxI -VI)	Ubiquitous	[46]	prdx-3	Mitochondria	[47]
			prdx-2	Intestine	[48]

Glutathione peroxidase (GPx1-8)	Ubiquitous	[49]	*gpx1-8 *	Unknown	[50]

Glutathione S-transferases (GSTs)	Cytosol Mitochondria Endoplasmic reticulum Nucleus Plasma membrane	[51]	*Ce*-GST-p24 (gst-4/*K08F4.7*),* CeGSTP2-2 *(*gst-10*) and* GSTO-1 *(C29E4.7)	Intestine Muscle cells Neurons	[52]

## References

[1] Whiting DR, Guariguata L, Weil C, Shaw J (2011). IDF Diabetes Atlas: global estimates of the prevalence of diabetes for 2011 and 2030. *Diabetes Research and Clinical Practice*.

[2] Pitocco D, Zaccardi F, di Stasio E (2010). Oxidative stress, nitric oxide, and diabetes. *The Review of Diabetic Studies*.

[3] Bashan N, Kovsan J, Kachko I, Ovadia H, Rudich A (2009). Positive and negative regulation of insulin signaling by reactive oxygen and nitrogen species. *Physiological Reviews*.

[4] Brownlee M (2005). The pathobiology of diabetic complications: a unifying mechanism. *Diabetes*.

[5] Naudi A, Jove M, Ayala V (2012). Cellular dysfunction in diabetes as maladaptive response to mitochondrial oxidative stress. *Experimental Diabetes Research*.

[6] C. elegans Sequencing Consortium (1999). Erratum: genome sequence of the nematode *C. elegans*: a platform for investigating biology (1998,282(5396):2012-8). *Science*.

[7] Hillier LW, Coulson A, Murray JI, Bao Z, Sulston JE, Waterston RH (2005). Genomics in *C. elegans*: so many genes, such a little worm. *Genome Research*.

[8] Harris TW, Chen N, Cunningham F (2004). WormBase: a multi-species resource for nematode biology and genomics. *Nucleic Acids Research*.

[9] Brenner S (1974). The genetics of *Caenorhabditis elegans*. *Genetics*.

[10] Le TT, Duren HM, Slipchenko MN, Hu C-D, Cheng J-X (2010). Label-free quantitative analysis of lipid metabolism in living *Caenorhabditis elegans*. *Journal of Lipid Research*.

[11] Hall D, Altun Z (2008). *C. Elegans Atlas*.

[12] Kimura KD, Tissenbaum HA, Liu Y, Ruvkun G (1997). daf-2, an insulin receptor-like gene that regulates longevity and diapause in *Caenorhabditis elegans*. *Science*.

[13] Kenyon C (2011). The first long-lived mutants: discovery of the insulin/IGF-1 pathway for ageing. *Philosophical Transactions of the Royal Society B: Biological Sciences*.

[14] Brown GC (1992). Control of respiration and ATP synthesis in mammalian mitochondria and cells. *The Biochemical Journal*.

[15] Vafai SB, Mootha VK (2012). Mitochondrial disorders as windows into an ancient organelle. *Nature*.

[16] Wallace DC (1999). Mitochondrial diseases in man and mouse. *Science*.

[17] Gao L, Laude K, Cai H (2008). Mitochondrial pathophysiology, reactive oxygen species, and cardiovascular diseases. *Veterinary Clinics of North America—Small Animal Practice*.

[18] Murfitt RR, Vogel K, Sanadi DR (1976). Characterization of the mitochondria of the free living nematode, *Caenorhabditis elegans*. *Comparative Biochemistry and Physiology B: Comparative Biochemistry*.

[19] Okimoto R, Macfarlane JL, Clary DO, Wolstenholme DR (1992). The mitochondrial genomes of two nematodes, *Caenorhabditis elegans* and *Ascaris suum*. *Genetics*.

[20] Li J, Cai T, Wu P (2009). Proteomic analysis of mitochondria from *Caenorhabditis elegans*. *Proteomics*.

[21] Grad LI, Lemire BD (2004). Mitochondrial complex I mutations in *Caenorhabditis elegans* produce cytochrome c oxidase deficiency, oxidative stress and vitamin-responsive lactic acidosis. *Human Molecular Genetics*.

[22] Yang W, Hekimi S (2010). Two modes of mitochondrial dysfunction lead independently to lifespan extension in *Caenorhabditis elegans*. *Aging Cell*.

[23] Wadsworth WG, Riddle DL (1989). Developmental regulation of energy metabolism in *Caenorhabditis elegans*. *Developmental Biology*.

[24] Mullaney BC, Ashrafi K (2009). *C. elegans* fat storage and metabolic regulation. *Biochimica et Biophysica Acta—Molecular and Cell Biology of Lipids*.

[25] Kuang J, Ebert PR (2012). The failure to extend lifespan via disruption of complex II is linked to preservation of dynamic control of energy metabolism. *Mitochondrion*.

[26] Dröge W (2002). Free radicals in the physiological control of cell function. *Physiological Reviews*.

[27] Murphy MP (2009). How mitochondria produce reactive oxygen species. *The Biochemical Journal*.

[28] Balaban RS, Nemoto S, Finkel T (2005). Mitochondria, oxidants, and aging. *Cell*.

[29] Trachootham D, Lu W, Ogasawara MA, Valle NR-D, Huang P (2008). Redox regulation of cell survival. *Antioxidants & Redox Signaling*.

[30] Kamata H, Hirata H (1999). Redox regulation of cellular signalling. *Cellular Signalling*.

[31] Davies KJA (2000). Oxidative stress, antioxidant defenses, and damage removal, repair, and replacement systems. *IUBMB Life*.

[32] Finkel T, Holbrook NJ (2000). Oxidants, oxidative stress and the biology of ageing. *Nature*.

[33] Fridovich I (1995). Superoxide radical and superoxide dismutases. *Annual Review of Biochemistry*.

[34] Fridovich I (1978). The biology of oxygen radicals. *Science*.

[35] Gems D, Doonan R (2009). Antioxidant defense and aging in *C. elegans*: is the oxidative damage theory of aging wrong?. *Cell Cycle*.

[36] Gems D (2009). Ageing and oxidants in the nematode *Caenorhabditis elegans*. *SEB Experimental Biology Series*.

[37] Doonan R, McElwee JJ, Matthijssens F (2008). Against the oxidative damage theory of aging: superoxide dismutases protect against oxidative stress but have little or no effect on life span in *Caenorhabditis elegans*. *Genes & Development*.

[38] Yen K, Patel HB, Lublin AL, Mobbs CV (2009). SOD isoforms play no role in lifespan in ad lib or dietary restricted conditions, but mutational inactivation of SOD-1 reduces life extension by cold. *Mechanisms of Ageing and Development*.

[39] Yanase S, Onodera A, Tedesco P, Johnson TE, Ishii N (2009). SOD-1 deletions in *Caenorhabditis elegans* alter the localization of intracellular reactive oxygen species and show molecular compensation. *The Journals of Gerontology A: Biological Sciences and Medical Sciences*.

[40] van Raamsdonk JM, Hekimi S (2009). Deletion of the mitochondrial superoxide dismutase sod-2 extends lifespan in *Caenorhabditis elegans*. *PLoS Genetics*.

[41] van Raamsdonk JM, Hekimi S (2012). Superoxide dismutase is dispensable for normal animal lifespan. *Proceedings of the National Academy of Sciences of the United States of America*.

[42] White JA, Scandalios JG (1989). Deletion analysis of the maize mitochondrial superoxide dismutase transit peptide. *Proceedings of the National Academy of Sciences of the United States of America*.

[43] Murakami K, Ichinohe Y, Koike M (2013). VCP Is an integral component of a novel feedback mechanism that controls intracellular localization of catalase and H_2_O_2_ levels. *PloS ONE*.

[44] Petriv OI, Rachubinski RA (2004). Lack of peroxisomal catalase causes a progeric phenotype in *Caenorhabditis elegans*. *The Journal of Biological Chemistry*.

[45] Taub J, Lau JF, Ma C (1999). A cytosolic catalase is needed to extend adult lifespan in *C. elegans* daf-C and clk-1 mutants. *Nature*.

[46] Schröder E, Brennan JP, Eaton P (2008). Cardiac peroxiredoxins undergo complex modifications during cardiac oxidant stress. *The American Journal of Physiology—Heart and Circulatory Physiology*.

[47] Ranjan M, Gruber J, Ng LF, Halliwell B (2013). Repression of the mitochondrial peroxiredoxin antioxidant system does not shorten life span but causes reduced fitness in *Caenorhabditis elegans*. *Free Radical Biology & Medicine*.

[48] Oláhová M, Taylor SR, Khazaipoul S (2008). A redox-sensitive peroxiredoxin that is important for longevity has tissue- and stress-specific roles in stress resistance. *Proceedings of the National Academy of Sciences of the United States of America*.

[49] Sena LA, Chandel NS (2012). Physiological roles of mitochondrial reactive oxygen species. *Molecular Cell*.

[50] Benner J, Daniel H, Spanier B (2011). A glutathione peroxidase, intracellular peptidases and the tor complexes regulate peptide transporter PEPT-1 in *C. elegans*. *PLoS ONE*.

[51] Raza H (2011). Dual localization of glutathione S-transferase in the cytosol and mitochondria: implications in oxidative stress, toxicity and disease. *The FEBS Journal*.

[52] Leiers B, Kampkötter A, Grevelding CG, Link CD, Johnson TE, Henkle-Dührsen K (2003). A stress-responsive glutathione S-transferase confers resistance to oxidative stress in *Caenorhabditis elegans*. *Free Radical Biology & Medicine*.

[53] Zamocky M, Furtmüller PG, Obinger C (2008). Evolution of catalases from bacteria to humans. *Antioxidants & Redox Signaling*.

[54] Larsen PL (1993). Aging and resistance to oxidative damage in *Caenorhabditis elegans*. *Proceedings of the National Academy of Sciences of the United States of America*.

[55] Schulz TJ, Zarse K, Voigt A, Urban N, Birringer M, Ristow M (2007). Glucose restriction extends *Caenorhabditis elegans* life span by inducing mitochondrial respiration and increasing oxidative stress. *Cell Metabolism*.

[56] Margis R, Dunand C, Teixeira FK, Margis-Pinheiro M (2008). Glutathione peroxidase family—an evolutionary overview. *The FEBS Journal*.

[57] Salinas AE, Wong MG (1999). Glutathione S-transferases—a review. *Current Medicinal Chemistry*.

[58] Burmeister C, Lüersen K, Heinick A (2008). Oxidative stress in *Caenorhabditis elegans*: protective effects of the Omega class glutathione transferase (GSTO-1). *FASEB Journal*.

[59] Ayyadevara S, Engle MR, Singh SP (2005). Lifespan and stress resistance of *Caenorhabditis elegans* are increased by expression of glutathione transferases capable of metabolizing the lipid peroxidation product 4-hydroxynonenal. *Aging Cell*.

[60] Tawe WN, Eschbach M-L, Walter RD, Henkle-Dührsen K (1998). Identification of stress-responsive genes in *Caenorhabditis elegans* using RT-PCR differential display. *Nucleic Acids Research*.

[61] Rhee SG, Yang K-S, Kang SW, Woo HA, Chang T-S (2005). Controlled elimination of intracellular H_2_O_2_: regulation of peroxiredoxin, catalase, and glutathione peroxidase via post-translational modification. *Antioxidants & Redox Signaling*.

[62] Isermann K, Liebau E, Roeder T, Bruchhaus I (2004). A peroxiredoxin specifically expressed in two types of pharyngeal neurons is required for normal growth and egg production in *Caenorhabditis elegans*. *Journal of Molecular Biology*.

[63] Gami MS, Wolkow CA (2006). Studies of *Caenorhabditis elegans* DAF-2/insulin signaling reveal targets for pharmacological manipulation of lifespan. *Aging Cell*.

[64] Brownlee M (2001). Biochemistry and molecular cell biology of diabetic complications. *Nature*.

[65] Giugliano D, Ceriello A, Paolisso G (1995). Diabetes mellitus, hypertension, and cardiovascular disease: which role for oxidative stress?. *Metabolism: Clinical and Experimental*.

[66] Kawamura M, Heinecke JW, Chait A (1994). Pathophysiological concentrations of glucose promote oxidative modification of low density lipoprotein by a superoxide-dependent pathway. *The Journal of Clinical Investigation*.

[67] Schlotterer A, Kukudov G, Bozorgmehr F (2009). *C. elegans* as model for the study of high glucose-mediated life span reduction. *Diabetes*.

[68] Hayes JD, McMahon M (2001). Molecular basis for the contribution of the antioxidant responsive element to cancer chemoprevention. *Cancer Letters*.

[69] Cuadrado A, Moreno-Murciano P, Pedraza-Chaverri J (2009). The transcription factor Nrf2 as a new therapeutic target in Parkinson’s disease. *Expert Opinion on Therapeutic Targets*.

[70] Moi P, Chan K, Asunis I, Cao A, Kan YW (1994). Isolation of NF-E2-related factor 2 (Nrf2), a NF-E2-like basic leucine zipper transcriptional activator that binds to the tandem NF-E2/AP1 repeat of the *β*-globin locus control region. *Proceedings of the National Academy of Sciences of the United States of America*.

[71] McMahon M, Itoh K, Yamamoto M, Hayes JD (2003). Keap1-dependent proteasomal degradation of transcription factor Nrf2 contributes to the negative regulation of antioxidant response element-driven gene expression. *The Journal of Biological Chemistry*.

[72] Kwak M-K, Wakabayashi N, Kensler TW (2004). Chemoprevention through the Keap1-Nrf2 signaling pathway by phase 2 enzyme inducers. *Mutation Research—Fundamental and Molecular Mechanisms of Mutagenesis*.

[73] Sykiotis GP, Bohmann D (2010). Stress-activated cap’n’collar transcription factors in aging and human disease. *Science Signaling*.

[74] Aleksunes LM, Reisman SA, Yeager RL, Goedken MJ, Klaassen CD (2010). Nuclear factor erythroid 2-related factor 2 deletion impairs glucose tolerance and exacerbates hyperglycemia in type 1 diabetic mice. *The Journal of Pharmacology and Experimental Therapeutics*.

[75] Kensler TW, Qian G-S, Chen J-G, Groopman JD (2003). Translational strategies for cancer prevention in liver. *Nature Reviews Cancer*.

[76] Yu Z, Shao W, Chiang Y (2011). Oltipraz upregulates the nuclear factor (erythroid-derived 2)-like 2 [corrected](NRF2) antioxidant system and prevents insulin resistance and obesity induced by a high-fat diet in C57BL/6J mice. *Diabetologia*.

[77] Xu J, Kulkarni SR, Donepudi AC, More VR, Slitt AL (2012). Enhanced Nrf2 activity worsens insulin resistance, impairs lipid accumulation in adipose tissue, and increases hepatic steatosis in leptin-deficient mice. *Diabetes*.

[78] An JH, Blackwell TK (2003). SKN-1 links *C. elegans* mesendodermal specification to a conserved oxidative stress response. *Genes & Development*.

[79] Tullet JMA, Hertweck M, An JH (2008). Direct Inhibition of the Longevity-Promoting Factor SKN-1 by Insulin-like Signaling in *C. elegans*. *Cell*.

[80] Kaletsky R, Murphy CT (2010). The role of insulin/IGF-like signaling in *C. elegans* longevity and aging. *DMM Disease Models and Mechanisms*.

[81] Honda Y, Honda S (1999). The daf-2 gene network for longevity regulates oxidative stress resistance and Mn-superoxide dismutase gene expression in *Caenorhabditis elegans*. *FASEB Journal*.

[82] Choe KP, Przybysz AJ, Strange K (2009). The WD40 repeat protein WDR-23 functions with the CUL4/DDB1 ubiquitin ligase to regulate nuclear abundance and activity of SKN-1 in *Caenorhabditis elegans*. *Molecular and Cellular Biology*.

[83] Blackwell TK, Bowerman B, Priess JR, Weintraub H (1994). Formation of a monomeric DNA binding domain by Skn-1 bZIP and homeodomain elements. *Science*.

[84] Back P, Matthijssens F, Vlaeminck C, Braeckman BP, Vanfleteren JR (2010). Effects of sod gene overexpression and deletion mutation on the expression profiles of reporter genes of major detoxification pathways in *Caenorhabditis elegans*. *Experimental Gerontology*.

[85] Park S-K, Tedesco PM, Johnson TE (2009). Oxidative stress and longevity in *Caenorhabditis elegans* as mediated by SKN-1. *Aging Cell*.

[86] Lapierre LR, Hansen M (2012). Lessons from *C. elegans*: signaling pathways for longevity. *Trends in Endocrinology and Metabolism*.

[87] Taguchi A, White MF (2008). Insulin-like signaling, nutrient homeostasis, and life span. *Annual Review of Physiology*.

[88] Li W, Kennedy SG, Ruvkun G (2003). daf-28 encodes a *C. elegans* insulin superfamily member that is regulated by environmental cues and acts in the DAF-2 signaling pathway. *Genes & Development*.

[89] Malone EA, Inoue T, Thomas JH (1996). Genetic analysis of the roles of daf-28 and age-1 in regulating *Caenorhabditis elegans* dauer formation. *Genetics*.

[90] Kenyon C, Chang J, Gensch E, Rudner A, Tabtiang R (1993). A *C. elegans* mutant that lives twice as long as wild type. *Nature*.

[91] Kenyon C (2005). The plasticity of aging: insights from long-lived mutants. *Cell*.

[92] Kharade SV, Mittal N, Das SP, Sinha P, Roy N (2005). Mrg19 depletion increases S. cerevisiae lifespan by augmenting ROS defence. *FEBS Letters*.

[93] Schroeder EA, Shadel GS (2012). Alternative mitochondrial fuel extends life span. *Cell Metabolism*.

[94] Ristow M, Zarse K, Oberbach A (2009). Antioxidants prevent health-promoting effects of physical exercise in humans. *Proceedings of the National Academy of Sciences of the United States of America*.

[95] le Bourg É (2009). Hormesis, aging and longevity. *Biochimica et Biophysica Acta*.

[96] Calabrese EJ, Baldwin LA (2002). Defining hormesis. *Human & Experimental Toxicology*.

[97] Yang W, Hekimi S (2010). A mitochondrial superoxide signal triggers increased longevity in *Caenorhabditis elegans*. *PLoS Biology*.

[98] Kolb H, Eizirik DL (2012). Resistance to type 2 diabetes mellitus: a matter of hormesis?. *Nature Reviews: Endocrinology*.

[99] Beckman KB, Ames BN (1998). The free radical theory of aging matures. *Physiological Reviews*.

